# Radiofrequency Lesioning for Movement and Psychiatric Disorders-Experience of 107 Cases

**DOI:** 10.3389/fnhum.2021.673848

**Published:** 2021-06-14

**Authors:** Paresh K. Doshi

**Affiliations:** Jaslok Hospital and Research Centre, Mumbai, India

**Keywords:** radiofrequency lesioning, pallidotomy, thalamotomy, subthalamotomy, anterior capsulotomy

## Abstract

**Background:**

Radiofrequency lesioning (RFL) though used since the 1950s, had been replaced by DBS in the 1990s. The availability of magnetic resonance-guided focused ultrasound for lesioning has renewed the interest in RFL.

**Objective:**

This paper analysis RFL in contemporary Functional Neurosurgery for various indications and its outcome. Complication rates of RFL are compared with the same author’s experience of DBS.

**Methods:**

One hundred and seven patients underwent RFL between 1998 and 2019. Indications included Parkinson’s Disease (PD), tremors, dystonia, and obsessive-compulsive disorders (OCD). The surgeries performed include thalamotomy (29), pallidotomy (49), subthalamotomy (23), and anterior capsulotomy/nucleus accumbens lesioning (6). Appropriate rating scales were used for preoperative and postoperative evaluations.

**Results:**

There was a 25% recurrence rate of tremors for PD after thalamotomy. Writer’s cramp rating scale improved from a mean of 10.54–1.6 in task specific dystonia (TSD) patients, after thalamotomy. In PD patients, after pallidotomy, contralateral motor Unified Parkinson’s Disease Rating Scale (UPDRS) and dyskinesia scores, improved by 41 and 57%, respectively, at 1-year. Burke-Fahn-Marsden Dystonia Rating Scale in hemidystonia patients improved from 18.04 to 6.91, at 1-year. There was 32 and 31% improvement in total and motor UPDRS, respectively, in the subthalamotomy patients, at 2-year. All patients of OCD were in remission. There were three deaths in the pallidotomy group. Postoperative, dysarthria, confusion, hemiparesis, dyskinesia, and paraesthesia occurred in 12 patients, of which, 7 were transient.

**Conclusion:**

RFL is a useful option in a select group of patients with tremors and dystonia. It is our preferred treatment option for TSD and OCD.

Radiofrequency lesioning (RFL) was introduced in 1953 when William Sweet showed that radiofrequency (RF) lesioning could provide a more precise and quantifiable lesion over direct current lesioning, which was being practiced during that period (Sweet and Mark, 1953). Over the subsequent years, with the progressive development in the technology of RF lesioning, stereotactic apparatus, CT and MRI scans, coupled with the knowledge and understanding of psychiatric and movement disorders, there was widespread utilization of lesioning in functional neurosurgery (Lozano et al., 2018). With the introduction of Deep Brain Stimulation (DBS) for tremors and later on for Parkinson’s Disease (PD) the practice of RF lesioning started declining rapidly (Rughani et al., 2013). The advent of magnetic resonance-guided focused ultrasound has once again revived the interest in lesioning surgeries (Franzini et al., 2020). We have been practicing lesioning surgeries in our functional neurosurgical practice along with DBS surgery for the last 22 years. We plan to review the indications and outcomes of these surgical procedures. Some of the salient features of each surgical procedure are also described.

## Methods

**FIGURE 1:**
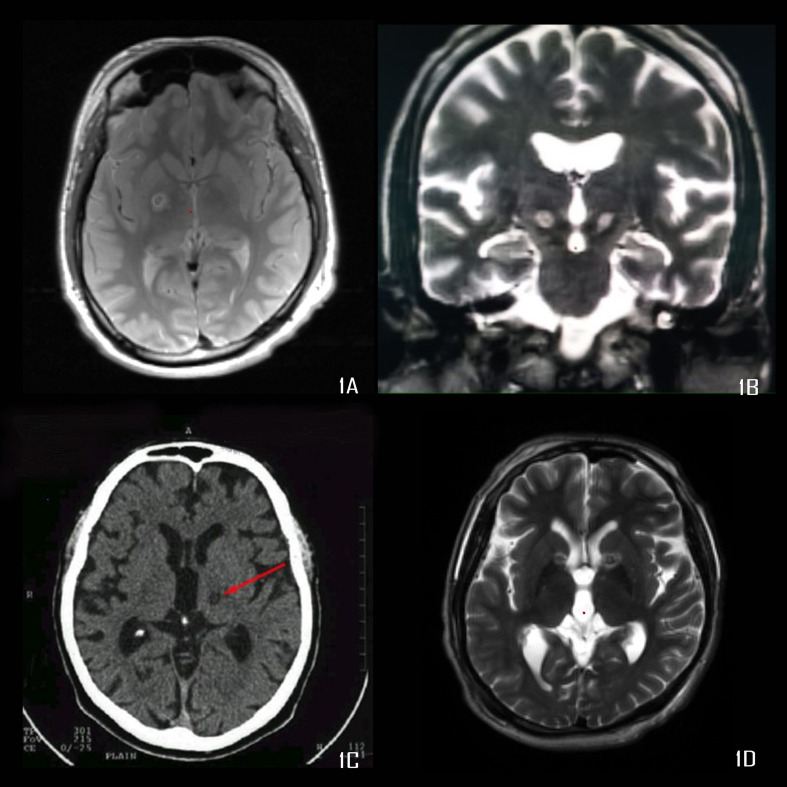
**(A)** Proton density image showing right pallidotomy, **(B)** T2 weighted MRI showing bilateral subthalamotomy, **(C)** CT scan showing Vim thalamotomy, **(D)** T2 weighted MRI showing bilateral anterior capsulotomy.

We retrospectively analyzed all the patients who underwent lesional surgery for movement or psychiatric disorders, from 1997 to 2019. We have performed 634 surgeries for movement and psychiatric disorders during this period, of which, 107 cases have undergone RF lesioning. All surgeries were done by the author. The study was approved by the IRB of our institute, through their letters dated 15/11/2013 and 23/6/2015. Table 1 shows the indications for which these surgeries were performed. The common surgical procedures performed included the Ventrointermedius (Vim) nucleus and Ventro-oral (Vo) nucleus of thalamus lesioning (Figure 1C), Globus pallidus internus (GPi) lesioning (Figure 1A), Subthalamic nucleus (STN) lesioning (Figure 1B), and anterior capsulotomy/Nucleus accumbens (NAc) lesioning (Figure 1D).

**TABLE 1:** Details of the indications and procedures performed.

	**Pallidotomy**		**Thalamotomy**		**Subthalamotomy**		**Anterior capsulotomy/nucleus accumbens lesioning**
	**Unilat**	**Bilat**	**Unilat**	**Bilat**	**Unilat**	**Bilat**	**Bilateral**
PD	31		9		4	19	
Generalized Dystonia		2					
Hemidystonia	16						
Focal hand dystonia			13				
Post stroke tremor			5				
Essential tremor			2				
OCD							6
Total	49		29		23		6

*PD, Parkinson’s disease; OCD, obsessive-compulsive disorder; Unilat, unilateral; Bilat, bilateral*.

### Patient Selection

#### Parkinson’s Disease

Patients with advanced PD and motor fluctuations were selected for pallidotomy. Detailed inclusion and exclusion criteria were similar to those described earlier (Kishore et al., 1997; Doshi et al., 2003). Pallidotomy was performed on the side contralateral to the most dyskinetic side, or the most affected side. Most of the pallidotomies for PD were performed between 1998 and 2004, as there was a limited experience with DBS. Some tremors dominant PD patients underwent thalamotomy (*N* = 9). Post-2004, as confidence in DBS, increased and the limitation of unilateral pallidotomy started being realized (Samii et al., 1999; Pal et al., 2000), we leaned more toward DBS. From 2004, we began offering STN lesioning instead of pallidotomy or thalamotomy for all PD patients who needed surgery but could not afford DBS. Initially, we offered unilateral subthalamotomy (*N* = 4), but later, we offered all patients bilateral subthalamotomy (*N* = 19). In select patients with an asymmetric disease (especially severe dyskinesia), who would need few years to arrange funds for DBS, we offer unilateral pallidotomy to tide over the period until they can make the arrangements (*N* = 6). This would make STN available as a target for DBS in the future. We had five such cases who first underwent pallidotomy and then bilateral STN DBS.

#### Tremors

Patients with uncontrolled tremors were offered a thalamotomy. All thalamotomies were unilateral. In the case of essential tremors (ET), it was performed contralateral to the dominant hand, to enable the patient to perform his activities of daily living. The preferred surgery for ET was bilateral DBS, but a thalamotomy was offered if the patient could not afford DBS. For all unilateral non-PD and ET indications, our first preference for surgery was thalamotomy.

#### Dystonia

For hemidystonia and generalized dystonia, we prefer DBS. For hemidystonia, we offer pallidotomy as an alternative to patients who cannot afford DBS. Pallidotomy is not offered for generalized dystonia. We had two exceptions to this, one was in the very early part of our practice (in 1999) and the second was as a palliative procedure for a patient in status dystonicus, who was not able to afford DBS. For the task specific focal dystonia, we prefer Vo thalamotomy. Again, the indications, selection criteria, and workup are similar to those described in the literature (Krauss, 2010).

#### Obsessive-Compulsive Disorders (OCD)

We prefer capsulotomy over DBS for OCD. The indications and selection criteria have been defined earlier (Doshi, 2011; Doshi et al., 2019). The important inclusion criteria included, refractory severe OCD (Yale-Brown Obsessive Compulsive Scale, Y-BOCS ≥ 28) (Goodman et al., 1989) and failure of three drugs (2 selective serotonin reuptake inhibitors and Clomipramine), and cognitive and behavior therapy to control the symptoms.

### Technique

The detailed technique for each surgery cannot be described, however, we will highlight salient features pertaining to RF lesioning and specific to our program.

#### Pallidotomy

Pallidotomy was performed using targeting based on MRI fused with stereotactic CT scan. We performed thin slices, T2 weighted, and inversion recovery coronal MRI perpendicular to the anterior commissure (AC)-posterior commissure (PC) plane. On the slice passing through the mammillary body, the pallidal target was chosen just above the lateral 1/3rd of the optic tract. This target was confirmed to be within the gray matter on the proton density axial MRI and T2 MRI. The entry point was planned just in the front of the coronal suture, 2.5–3.0 cm lateral to the midline. It was ensured that the trajectory point at the level of AC-PC was within the posterior 1/3rd of the GPi and 2–3 mm medial to the internal capsule. If not, the laterality of the target or the trajectory would need to be adjusted to achieve that. We performed microelectrode recording (MER) to define the boundaries of globus pallidus externus and GPi. The recording was usually started 10 mm above the target and was stopped 1 mm above the target. This was to avoid any damage to the optic tracts. Macro stimulation was performed to look for improvement and side effects. We usually performed 3 tract recordings; central, posterior, and medial, which helped us to define the posterior and medial boundaries of GPi. Once we confirm the target, we use a 2 × 2 mm exposed tip Radionics^®^ electrode to make lesions starting at the target and progressing dorsally upto 4 mm above the target in an increment of 2 mm steps. After each lesion, the improvement in rigidity and tremors was confirmed. Each lesion was made @ 70°C for 60 s. A minimum of two lesions and a maximum of three lesions were made.

#### Thalamotomy

Thalamotomy was performed using CT scan-based stereotactic targeting. The Vim target was between 2/10th and 3/10th of AC-PC length; anterior to PC and 50% of AC-PC length lateral to the midline. The laterality of the co-ordinate was further refined if the third ventricle was dilated (50% of the width of the third ventricle was added to the laterality co-ordinate. The z-coordinate of the target would be at the AC-PC plane. MER was used to locate the tremor cells. The somatotopic arrangement of the sensory thalamus was used as a guide to define the laterality of the target in the Vim (Kelly, 1980). Tremor control was assessed by stimulating @ 130 Hertz and 60 μs. If we obtain paraesthesia at the stimulation of less than 1 volt, we consider that we are very close to the sensory thalamus and move over the target anteriorly. The typical voltage for tremor control should be between 1 and 2 volts. At the same time, the patient should feel minimal paraesthesia in the region of interest. Once again, based on the somatotopy of the thalamus, the laterality is adjusted if the paraesthesia is in a different body part. The lesioning is performed using a Radionics^®^ 1 × 1 mm exposed tip electrode, starting the first lesion at the AC-PC plane, and moving dorsally in an increment of 1 mm to make three lesions. The lesion may be expanded if the tremors are not 100% controlled. Each lesion is made @ 70°C for 60 s. A similar procedure is followed for Vo lesioning (Doshi et al., 2017). The co-ordinates for the Vo lesioning were, 2 mm posterior to mid AC-PC point, 13.5 mm lateral and 1 mm above the AC-PC plane.

#### Subthalamotomy

The targeting and exploration of the subthalamic nucleus are similar to that for DBS (Doshi et al., 2003). This includes MER and macrostimulation to map the STN nucleus. The lesioning electrode used for subthalamotomy is 1 × 0.75 exposed tip. Two to three lesions are performed in the STN based on the length of the MER obtained. Here, once again, we use tremor control and rigidity reduction to judge the adequacy of the lesion. The number of lesions performed in our series has ranged from a minimum of 2 to a maximum of 3. The lesions were repeated at 1 mm increments. Each lesion is made @ 70°C for 60 s.

#### Anterior Capsulotomy/Nucleus Accumbens Lesioning

Our initial target was the junction of middle and posterior one-third of the anterior limb of the internal capsule based on the capsulotomy literature (Eljamel, 2008; Doshi, 2011). Over a period of time, based on the data of outcome of DBS for OCD, we moved our target more posteriorly and inferiorly. In the last two cases we lesioned the NAc (Nuttin et al., 2003; Denys et al., 2010). We used a 2 × 2 mm exposed tip probe for lesioning. The lesioning is made @ 70°C for 60 s. For initial capsulotomy, we made extensive lesions (three lesions with 2 × 2 electrode) (Doshi, 2011). However, the lesions in NAc were much smaller, comprising of only a single lesion by a 2 × 2 mm electrode.

### Statistics

Mean, Standard Deviations, and Standard errors (SE) were estimated for the pre-operative and post-operative scores of all tests. Student t-test was used to calculate statistical significance between preoperative and follow-up assessments. A *p*-value of <0.05 was considered statistically significant.

## Results

One hundred and seven patients underwent various lesioning procedures from 1997 to 2019. The age range for the group undergoing lesioning surgery was 17–72 years with a mean age of 45. Seventy-eight percent of the patients were male. All the patients undergoing surgery for task specific dystonia (TSD) and obsessive-compulsive disorder (OCD) were males. The improvement was variable based on the disease and the surgery involved. Table 2 summarizes the overall improvement observed in these series.

**TABLE 2:** Surgical outcome following radiofrequency lesioning.

	**Procedure**	**Preoperative scores**	**Postoperative scores**	**% improvement**
PD (tremors)	Thalamotomy	4.3	1.1	74.42
PD (contralateral motor UPDRS)	Pallidotomy	22.6	11.4	49.56
PD (total UPDRS)	Subthalamotomy	81	50.6	37.53
PD (motor UPDRS)	Subthalamotomy	54.5	35	35.78
Hemidystonia (BFMDRS)	Pallidotomy	18.04	6.91	61.70
Focal hand dystonia (WCRS)	Voa thalamotomy	10.54	1.6	84.82
OCD (Y-BOCS)	AC, NAc	36.5	8.17	77.62
OCD (HAM-A)	AC, NAc	26.17	4.17	84.07
OCD (HAM-D)	AC, NAc	27.67	5.33	80.74

*PD, Parkinson’s disease; OCD, obsessive-compulsive disorder; AC, anterior capsulotomy; NAc, nucleus accumbens; UPDRS, Unified Parkinson’s disease rating scale; BFMDRS, Burke-Fahn-Marsden Dystonia Rating score; WCRS, writer’s cramp rating scale; Y-BOCS, Yale-Brown obsessive compulsive scale; HAM-A, Hamilton anxiety rating scale; HAM-D, Hamilton depression rating scale.*

### Thalamotomy

The outcome of thalamotomy for tremors and TSD have been separately analyzed. The follow-up ranged from 6 months to 2 years. If the patients were symptom-free, we would discharge them from follow-up.

#### Tremor Results

In PD patients, the mean tremor scores (item 20 and 21 of UPDRS, for the affected side; minimum = 0 and maximum = 8) improved from a mean of 4.3–1.1 (*p* = 0.0005). We had a good tremor control in 7 of the 9 patients. Two patients had a recurrence of tremors within the first 3 months. Both ET patients had complete control of tremors and four out of five post-stroke patients were free of tremor, i.e., there was no residual tremors.

#### TSD Results

Nine of the thirteen patients of TSD had complete resolution of symptoms (Writer’s cramp rating scale, WCRS 0–2) (Wissel et al., 1996). Four patient had minor residual symptoms. The WCRS improved from a mean of 10.54 (range 4–28, SE 2.17) to a mean of 1.6 (range 0–6, SE 0.68), at the last follow-up (range 19–93 months, mean 57 months). This was statistically significant (*p* < 0.0001).

### Pallidotomy

The results of pallidotomy were also separately analyzed based on the indication, as they have different outcomes.

#### Pallidotomy for PD

We had 28 cases of pallidotomies for PD, followed up at an interval of 6 months and 1 year. Unified Parkinson’s Disease Rating Scale version 3.0 was used to assess the outcome of PD patients (Fahn et al., 1987). Preoperative, total UPDRS changed from a mean of 90 (range 79–121, SE 12.01) to a mean of 66 (range 62–93, SE 19.17) at 6 months and a mean of 72 (range 16–108, SE 11.7). This was statistically not significant (*p* = 0.50 and 0.15 at 6 and 12 months, respectively). Similarly, the preoperative motor UPDRS (UPDRS part III, item 18–31) changed from a mean of 63 (range 40–96, SE 10.88) to a mean of 44 (range 9–80, SE 14.15) at 6 months and a mean of 48 (range 37–57, SE 2.89) at 12 months. This too was statistically not significant improvement (*p* = 0.05 and 0.15 at 6 and 12 months, respectively).

The contralateral motor UPDRS improved from the preoperative mean score of 22.6 (range 15–29, SE 2.03) to a mean score of 11.4 (range 0–20, SE 2.63, *p* < 0.0001), at 6 months and a mean score of 13.29 (range 6–20, SE 2.15, *p* = 0.02), at 1-year follow-up. The dyskinesia scores (UPDRS item 32–34) also showed an improvement, from a preoperative score mean of 6.6 (range 4–11, SE 1.17) to a mean of 2.4 (range 0–7, SE 1.29), and 2.86 (range 1–6, SE 0.63) at 6 and 12 months. This was statistically significant.

**FIGURE 2:**
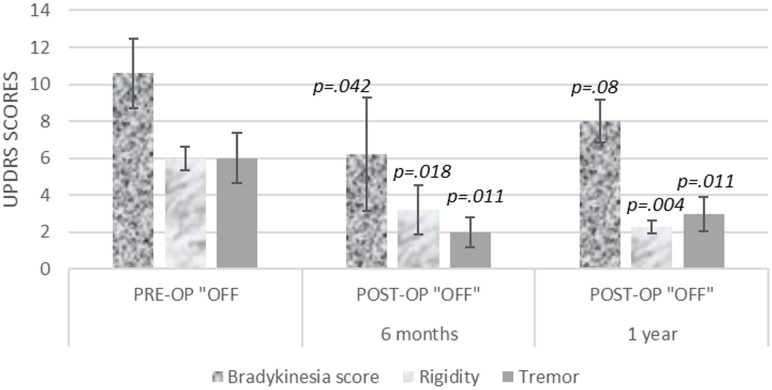
Contralateral bradykinesia, rigidity, and tremor UPDRS scores, at 6 months and 1 year after unilateral pallidotomy.

When contralateral, bradykinesia, rigidity, and tremors were evaluated, the tremor and rigidity scores showed sustained benefit, but the bradykinesia scores improvement did not reach up to statistical significance (Figure 2).

The activities of daily living scores (part II of UPDRS) and axial scores including arise from chair, gait, speech, and postural stability did not show statistically significant improvement at 6 or 12 months.

#### Pallidotomy for Hemidystonia

Patients with hemidystonia undergoing unilateral pallidotomy had a mixed outcome. The etiology for hemidystonia included stroke, encephalitis and head injury. One-year follow-up was available for 12 patients. Four patients had complete resolution of dystonia (BFMDRS 0–2), seven had variable improvements ranging from 30 to 70% reduction in the Burke-Fahn-Marsden Dystonia Rating scores (BFMDRS) and Dystonia disability scale (DDS) (Burke et al., 1985). One patient had no improvement. The BFMDRS for the group improved from a mean of 18.04 (range 12–31.5, SE 2.54) to 6.91 (range 0–28, SE 2.34) (*p* < 0.0001) and DDS improved from a mean of 7.83 (range 4–13, SE 1.09) to 3.55 (range 0–11, SE 1.08) (*p* = 0.0002). A 17-year-old boy, suffering from Neurodegeneration with brain iron accumulation, who underwent bilateral pallidotomy, had improvement in status dystonicus.

### Subthalamotomy

**FIGURE 3:**
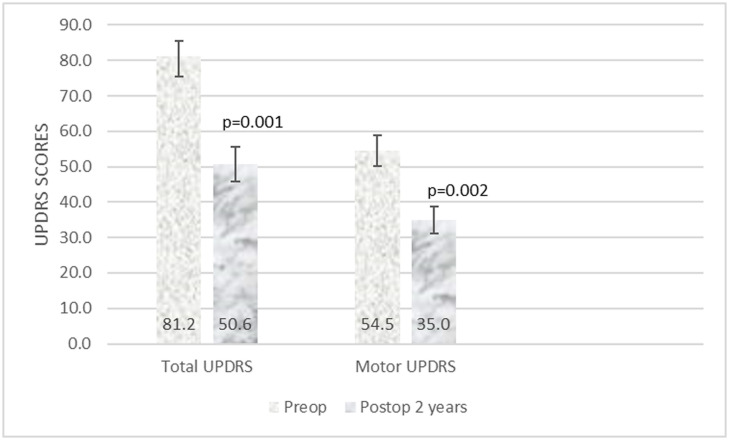
Total and motor UPDRS at two years follow-up after subthalamotomy.

We had a follow-up of only 11 patients, out of 19, who had undergone bilateral subthalamotomy. The reasons for this were multifactorial. Most of the patients came from distant villages and were not contactable. As they had limited resources, they did not come back for a follow-up and hence the detailed assessment was not available. The details of their off-period scores at 2 years follow-up are presented in Figure 3. There was a 32 and 31% improvement in the total and motor UPDRS scores. The total score improved from a mean of 81 (range 45–105, SE 5.87) preoperatively to 50.6 (range 32–89, SE 4.94) at follow up, this was statistically significant (*p* = 0.001). The motor UPDRS (UPDRS part III) improved from a mean of 54.5 (range 27–74, SE 4.31) to 35 (range 22–54, SE 3.8). This too was statistically significant (*p* = 0.002). We found differential motor improvement in 6/10 patients, with one side improving more than the other. The average levodopa equivalent dose was reduced from 823 to 661 mg.

### Anterior Capsulotomy (AC)/Nucleus Accumbens Lesioning

Four patients underwent AC and 2 patients underwent NAc lesioning (Table 3). The follow-up ranges from a minimum of 32 to 74 months. The Y-BOCS improved from a mean of 36.5 (range 26–40, SE 2.30) to 8.2 (range 0–12, SE 1.76); Hamilton Anxiety socres improved from a mean of 26.2 (range17–35, SE 2.6) to 4.2 (range 0–8, SE 1.6) and Hamilton Depression scores improved from a mean of 27.7 (range 13–39, SE 3.82) to 5.3 (range 3–12, SE 1.48) at the last follow-up. These all were statistically significant (Y-BOCS *p* = 0.0001, HAM-A *p* = 0.001, HAM-D *p* = 0.001). According to the criteria defined by Pallanti and Quercioli (2006), all patients were in remission.

**TABLE 3:** Data of patients undergoing anterior capsulotomy or NAc lesioning for OCD.

	**Surgery target**	**Preoperative scores**			**Follow up duration Y-BOCS (months)**	**Post-operative scores**		
		**YBOCS**	**HAM-A**	**HAM-D**		**YBOCS**	**HAM-A**	**HAM-D**
Case 1	AC	40	26	23	66	0	0	6
Case 2	AC	40	31	13	74	11	0	1.5
Case 3	AC	39	35	36	54	8	2	4.5
Case 4	AC	40	17	29	84	8	8	3
Case 5	NAc	26	26	26	44	10	8	12
Case 6	NAc	34	22	39	32	12	7	5

*AC, anterior capsulotomy; NAc, nucleus accumbens; Y-BOCS, Yale-Brown obsessive-compulsive scale; HAM-A, Hamilton anxiety rating scale; HAM-D, Hamilton depression rating scale*.

### Adverse Events

There was no mortality in thalamotomy, subthalamotomy and AC/NAc lesioning. There were three mortalities in pallidotomies in the perioperative period. There were two deaths in the PD group; one occurred 3 days after surgery (due to aspiration pneumonia) and another 3 weeks after surgery. Both of these patients were elderly. Following these observations, we decided not to offer lesional surgeries in patients above 70 years. One patient of neurodegeneration with brain iron accumulation, for whom we performed bilateral pallidotomy for status dystonicus, could not be weaned off the ventilator and succumbed to septicemia.

One patient developed transient hemiparesis and one patient dysarthria following thalamotomy.

In patients with pallidotomy, three patients developed hemiparesis of which two recovered and one had a permanent deficit. There was a hemorrhage at the site of pallidotomy in the patient with permanent hemiparesis. Two persons developed postoperative confusion.

In patients undergoing subthalamotomy, one patient developed unilateral dyskinesia, which subsided in 6 months. Two patients developed dysarthria and one patient developed paresthesia on one side of the body.

There was transient apathy noted in one patient undergoing anterior capsulotomy.

## Discussion

RF lesioning has been a preferred method for intracranial lesioning. Besides, movement disorders and psychiatric disorders surgeries, it has also been used in pain and epilepsy (Sharim and Pouratian, 2016; Voges et al., 2018). Lesion size is dependent on the size (diameter) of the electrode, length of the exposed tip, temperature, and duration of the lesion (Cosman et al., 1983). The size and shape of all intracranial targets are different, e.g., Vim is only 4 mm in width. Selecting the correct size of the lesioning electrode is essential for a successful circumscribed lesion. Using a 2 mm size electrode for lesioning Vim will form an elliptical lesion with a diameter of more than 8 mm (depending on temperature and time), posing a risk for lesioning adjacent structures (Cosman et al., 1983). Similarly, GPi and anterior capsulotomy require a larger volume of lesioning and hence bigger electrodes. Therefore, it is very important to use an electrode of the correct size for lesioning different nuclei.

Pallidotomy for PD was used during 1998–2004. The outcome was in line with that reported in the literature (Merello et al., 1999; Samii et al., 1999; Parkin et al., 2002). The ipsilateral improvement seen was transient, but the contralateral improvement in rigidity, dyskinesia, and tremor score significant and lasting. Due to the increased risk of irreversible complications, we do not offer any lesional surgeries to patients above 70 years. There have been few reports of bilateral pallidotomy with good motor improvements, suggesting a possible alternative for advanced PD (De Bie et al., 2002; York et al., 2007). Because of the limitations of subthalamotomy discussed below, we are considering exploring a staged bilateral pallidotomy as an alternative lesional procedure for PD.

Secondary hemidystonia is a challenge due to its heterogenicity. Due to its uncommon occurrence, limited information exists about the outcome of surgical intervention. This is the largest series of lesional surgeries reported for hemidystonia to date. Kim et al. (2012) found 73.2 and 75% improvement in BFMDRS and disability scale in four patients, undergoing DBS at 2-year follow-up. Starr and colleagues reported three patients undergoing pallidal DBS for postinfarct hemidystonia. They did not find significant improvement of BFMDRS in any patient at 1-year follow-up, but they note that there was a subjective and partial improvement, not captured by the BFMDRS scores. Pallidotomy for dystonia yielded similarly mixed results. Eltahawy et al. (2004) did not find any improvement after pallidotomy in three cases of secondary hemidystonia. In some patients there was a partial improvement, but significant improvement in pain. Chuang et al. (2002) reported benefit in five of the six patients; including two patients with repeat procedures; following thalamotomy for hemidystonia. In our series, we had similarly mixed responses. We also found that benefits cannot be always captured in the BFMDRS scales. On a longer follow-up, there was worsening in some patients and hence longer follow-up data may be useful. Though we would prefer to do pallidal DBS for unilateral dystonia, if the patient cannot afford the cost of the surgery or has difficulty in follow-up, we consider pallidotomy as the second-best option.

Thalamotomy is a very rewarding surgery for unilateral tremors of almost all etiologies. Intraoperative mapping of the thalamus and the size of the lesioning electrode (as discussed above) is critical to success. We only offered thalamotomy to certain PD patients, from 1998 to 2004, as later, we offered STN lesioning to similar patients with advanced PD.

Our treatment of choice for TSD has been Vo thalamotomy. The bias can be explained by the excellent results that have been obtained (Doshi et al., 2017). These results are similar to that observed by others (Horisawa et al., 2019).

STN lesioning has been a challenge for us. We had to titrate between 2 and 3 lesions on each side, based on the anatomy of the STN. Despite this, we had variable outcomes on each side even amongst the same individuals. Our improvement of 31% of motor UPDRS scores was much less than that of Alvarez et al. (2005), who reported 49% improvement. This could be attributed to the differential benefit between the right and the left side in six patients. Lesioning of the STN is not as widely understood as thalamotomy and pallidotomy. We remained cautious in making our lesion, using the smallest possible lesioning electrode, which could be one of the reasons for having lesser improvement. Our cautious approach is also reflected in the reduction of the number of patients suffering from hemiballism (1 out of 23) as compared to Su and Tseng (2002, 3/12) and Alvarez et al. (2005, 3/18).

Our first capsulotomy was performed, based on the experience of Lippitz et al. (1997) at the junction of posterior and middle third of the internal capsule, which was a large lesion. Though this patient had complete remission from OCD, he developed apathy. Later on, following the observations of Greenberg et al., we moved our capsulotomy target more posterior and inferior (Figure 1D; Greenberg et al., 2010). In the last two cases we lesioned NAc with an improved understanding of the role of NAc and evolution of the targeting in this region, for OCD (Sturm et al., 2003; van Kuyck et al., 2007; Greenberg et al., 2010). In a meta-analysis of OCD outcome following capsulotomy, Pepper et al., showed that 49% had a useful response (responders) and 20% had remission. Their criteria for deciding this was based on the Y-BOCS being less than 8 for consideration of remission. However, this is not what is generally accepted and the criteria considered for remission by us and others is Y-BOCS < 16 (Pallanti and Quercioli, 2006; Ruck et al., 2008; Alonso et al., 2015). In our series, all patients went into remission. We had a very rewarding outcome for which we do not have any explanation, accept all our patients had the type of obsessions, e.g., religious and sexual obsessions, checking, hand washing, etc., that are known to respond favorably to surgical intervention (Alonso et al., 2015). Christensen et al. (2002) also reported a similar outcome in two cases of capsulotomy. We have found that the improvement in symptoms takes an average of 6 months and sometimes has to be supplemented with aggressive medical and behavioral therapy.

Alternative lesioning procedures have also shown similar outcome. The Charlotsville group reported marked clinical improvement in 4 out of 5 patients undergoing gamma knife anterior capsulotomy for OCD (Sheehan et al., 2013). The Karolinska group reported improvement from a preoperative Y-BOCS score of 33.4–14.2 at a mean follow-up of 136.8 months, in nine patients undergoing gamma knife capsulotomy. They found that there was no significant difference between patients undergoing thermocapsulotomy and gamma knife capsulotomy (Ruck et al., 2008). In a series of 73 patients undergoing gamma knife thalamotomy for essential tremors, Niranjan et al. (2019) found 72% patients improved in all tremor scores at a median of 28 months of follow-up. Schlesinger et al. (2015) reported, that the Item 20 (maximum 4) scores improved from a mean of 2.7–0.0, in seven PD patients undergoing MRI guided focused ultrasound thalamotomy for PD. A multicenter randomized study of MRI guided focused ultrasound thalamotomy for essential tremor noted, 68% improvement in tremor scores in seventy six patients at 1 year follow-up (Elias et al., 2016).

One of the concerns about lesional surgery is the increased risk of complications. In this series, the overall complication rate was 14% and the permanent complication rate in 4% of the patients. Hariz and De Salles (1997) also found a permanent complication rate of 10% in their series. When we compared the results with our DBS series, the surgical complications were seen in 8.9% of the patients and hardware complications in an additional 5% of the patients, which are higher than the lesional surgeries (Doshi et al., 2021). Blomstedt and Hariz (2006) found perioperative complication rate to be the same in the patients undergoing DBS or lesional procedure. In another study, covering multiple centers across the United States and 4,961 DBS and 451 lesional surgery cases, Rughani et al. (2013) did not find any difference in mortality, perioperative complications, or hemorrhage between the two groups. We had no mortality in the DBS group. However, as mentioned earlier, our mortality in patients with pallidotomy was in the very early period of our experience, which we subsequently corrected and avoided any further mortality. There is a learning curve for all procedures and the complications decrease as the experience increases (Doshi et al., 2021). We had the most complications in the first 10 years of our practice when we had 67% of the complications. Careful patient selection, planning, and surgical technique are essential for minimizing complications.

## Conclusion

In our practice, we find RFL a useful functional neurosurgical option, and in certain cases, use it as a first choice of treatment, e.g., OCD and TSD. It should be practiced with the same or even more care than DBS if complications are to be minimized.

## Data Availability Statement

The raw data supporting the conclusions of this article will be made available by the authors, without undue reservation.

## Ethics Statement

The studies involving human participants were reviewed and approved by Institutional Review Board of Jaslok Hospital and Research Centre, Mumbai. The patients/participants provided their written informed consent to participate in this study.

## Author Contributions

PD conceptualized, drafted, and edited the entire manuscript, was the only surgeon who has performed all the above surgeries.

## Conflict of Interest

The author declares that the research was conducted in the absence of any commercial or financial relationships that could be construed as a potential conflict of interest.
